# Editorial: Inclusive schools for a diverse world: Psychological and educational factors and practices harming or promoting inclusion at school

**DOI:** 10.3389/fpsyg.2022.1049129

**Published:** 2022-10-12

**Authors:** Sabine Pirchio, Francesco Arcidiacono, Ylenia Passiatore

**Affiliations:** ^1^Sapienza University of Rome, Rome, Italy; ^2^Department of Dynamic and Clinical Psychology, and Health Studies, Rome, Italy; ^3^Research Department, Haute École Pédagogique BEJUNE, Biel/Bienne, Switzerland; ^4^Dipartimento di Scienze della Formazione, Roma Tre University, Rome, Italy

**Keywords:** inclusive education, teachers, social inclusion, special educational needs, learning difficulties, students

Inclusion is for schools a primary goal in providing instruction and education (Daiute et al., 2021) in an equal and fair learning environment for every student. Since the Salamanca Statement and Framework for Action on Special Needs Education (UNESCO, 1994), all the educational systems were called to implement some sort of inclusive actions in their schools to make them open to the diversity of the pupils and equipped to meet their social and academic needs. This implied important intended consequences on the educational practices put in action at school, on the non-discriminatory nature of the society, and on the economic sustainability of educational systems (Ainscow et al., 2019). The Salamanca agreement framed many important changes in the way pupils are involved and supported in education.

However, inclusive education is not a clearly defined construct, and it is differently conceptualized and implemented in various educational contexts of several countries, implying diverse attitudes toward it by different stakeholders (Krischler et al., 2019; Leijen et al., 2021).

A number of definitions of inclusion can be observed (Göransson and Nilholm, 2014), where it can be described by simply placing students with disabilities or with special needs in general education classrooms (*placement* definition), providing resources for the social and academic needs of students with disabilities or with special needs (*specified individualized* definition) or of all students (*general individualized* definition), or by creating communities with specific characteristics (*community* definition). In this regard, attitudes toward the students with special education needs (SEN) play an important role in influencing the creation of an inclusive school.

This Research Topic includes articles discussing the different factors and variables harming or promoting social and academic inclusion in a variety of contexts and involving diverse stakeholders.

The first factor characterizing the studies here presented is the “who should be included”, in other words the specificity of the students benefiting from the inclusion. An importantly targeted population, particularly in research in the last 10 years, is that of students who are migrant or from ethnic minorities (Costa et al.; Gitschthaler et al.; Wang; Glock et al.); their inclusion in general classroom would require teachers with intercultural competences and resources to overcome the eventual communication difficulties in the school language and to valorize the cultural richness of the classroom. Other articles focus on students with disabilities (Alvarez-Delgado et al.; Al Jaffal) or with special educational needs (Casino-García et al.; Lindner et al.; Kobs et al.). Noteworthily, several articles address inclusion of all the students, such as the works proposed by Liu et al., Schellenberg et al., Kivirand et al., and Graham et al..

The question of what factors influence inclusion at school may find an answer in different features of the experience and relationships in educational contexts. The papers included in this Research Topic confirm the important role of the teachers' attitudes (such as in Costa et al.; Kobs et al.; Glock et al.), as well as students' attitudes (Alvarez-Delgado et al.; Graham et al.).

Another important factor is represented by the teacher competence, in terms of multiculturality as it is discussed by Wang, and the question of how to teach students with specific disabilities (Al Jaffal). In this regard, studies investigating specific pedagogical interventions may give a valuable contribution to teacher training programs: the studies by Casino-García et al., Lindner et al., Gitschthaler et al., and Schellenberg et al. highlight the potential of curricular and extra-curricular strategies to support inclusion.

Finally, important differences may be observed in the implementation of inclusion according to the qualities of the school social context, as shown in the studies proposed by Liu et al., Kivirand et al., and Al Jaffal.

It is important that research in education not only investigates factors influencing the inclusion in schools but also tests and suggests practices and tools to promote inclusion. The studies presented in this Research Topic provide valuable indications and propose that teachers' training should play an important role rising the teachers' (and other school professionals') multicultural awareness, changing their attitudes and supporting equal and inclusive educational practices (Costa et al.; Wang; Glock et al.; Kivirand et al.). In addition, an intervention at the school level may increase important resources such as cultural services and school facilities, as showed by the studies of Liu et al., Gitschthaler et al., and Al Jaffal. Moreover, students are important actors in the scene of school inclusion: using collaborative students' practices (Casino-García et al.; Lindner et al.; Schellenberg et al.) and extracurricular activities in order to increase empathy and awareness (Alvarez-Delgado et al.) can contribute to changing the social climate of the classrooms.

In conclusion, we highlight the necessity to integrate the perspectives of different actors and stakeholders (in particular, teachers, students and families) to foster collaboration among different microsystems of the ecological environment where the students develop their competences and identity (Lindner et al.).

## Author contributions

All authors listed have made a substantial, direct, and intellectual contribution to the work and approved it for publication.

## Conflict of interest

The authors declare that the research was conducted in the absence of any commercial or financial relationships that could be construed as a potential conflict of interest.

## Publisher's note

All claims expressed in this article are solely those of the authors and do not necessarily represent those of their affiliated organizations, or those of the publisher, the editors and the reviewers. Any product that may be evaluated in this article, or claim that may be made by its manufacturer, is not guaranteed or endorsed by the publisher.
